# Prediction of death by neurologic criteria in patients with non-traumatic acute brain injury: a systematic review and meta-analysis

**DOI:** 10.1186/s12883-026-05503-2

**Published:** 2026-10-22

**Authors:** Daniela Schoene, Marie Nicklas, Eyad Altarsha, Norma J. Diel, Anne Trabitzsch, Konrad Pleul, Hagen B. Huttner, Kristian Barlinn

**Affiliations:** 1 Department of Neurology, Faculty of Medicine and University Hospital Carl Gustav Carus, Technische Universität Dresden, Dresden, Germany; 2 Department of Surgery, Faculty of Medicine and University Hospital Carl Gustav Carus, Technische Universität Dresden, Dresden, Germany; 3 Deutsche Stiftung Organtransplantation (DSO), Frankfurt am Main, Germany

**Keywords:** Death by neurologic criteria, Brain death, Prediction, Acute brain injury

## Abstract

**Background:**

Robust predictors of death by neurologic criteria (DNC) after non-traumatic acute brain injury remain insufficiently defined. We therefore aimed to systematically synthesize the available evidence on prediction of DNC in non-traumatic brain injury.

**Methods:**

We conducted a systematic review and meta-analysis in accordance with PRISMA guidelines. MEDLINE, Embase, Scopus, the Cochrane Library and Web of Science were searched to August 31, 2026. Eligible studies included randomized controlled trials, observational studies, case series and registry data. Studies were included in the quantitative synthesis when sufficient comparable data for the same prognostic factor were available from at least two studies. Pooled effect estimates were calculated using random-effects models. Risk of bias was assessed using the Quality in Prognosis Studies (QUIPS) tool. The certainty of evidence was assessed using the GRADE approach.

**Results:**

The database search identified 2,091 records. Eighteen studies comprising reported study cohorts of 9,070 participants were included. Quantitative synthesis included four studies (*n*=1,738), comprising two post-cardiac arrest and two stroke cohorts. In stroke populations, CT markers of severe intracranial mass effect were associated with a more than threefold increased odds of DNC (pooled OR 3.14, 95%CI 1.98–4.96; *p*<0.0001; I2=0%), while in post-cardiac arrest cohorts lower gray-white matter ratio values were associated with progression to DNC (pooled mean difference -0.15, 95%CI -0.22 to -0.07; *p*<0.001; I2=72%).

**Conclusions:**

Evidence on predictors of DNC after non-traumatic acute brain injury remains limited and methodologically heterogeneous, with only a small number of studies providing robust analyses. Across these studies, CT-based markers - particularly indicators of diffuse cerebral edema or intracranial mass effect - show the most reproducible associations with progression to DNC.

**Supplementary Information:**

The online version contains supplementary material available at https://doi.org/10.1186/s12883-026-05503-2.

## Introduction

Death by neurologic criteria (DNC) is defined as the complete and irreversible loss of all brain function, including the brainstem, and is recognized as both a medical and legal determination of death. While the diagnosis of DNC is governed by established protocols, no standardized approach exists for the identification of patients at risk of progressing toward DNC. In clinically unresponsive patients with severe brain injury, decisions to withdraw life-sustaining therapy substantially contribute to mortality, underscoring the importance of accurate prognostication in the disease course [1].

Advances in neurointensive care have reduced mortality and improved functional outcomes in patients with acute brain injury. Nevertheless, clinical trajectories remain highly heterogeneous, ranging from neurological recovery to persistent disorders of consciousness or progression to DNC. Early and reliable prognostic information is essential for timely clinical decision-making, appropriate allocation of intensive care resources and structured communication with families. However, prognostication in unresponsive patients - particularly in the post-injury phase after events such as cardiac arrest - remains limited by considerable uncertainty [1, 2].

Numerous prognostic scores, imaging findings and biomarkers have been developed to predict neurological outcomes after acute brain injury [1–3]. Although widely used for general outcome assessment, these tools are not specifically designed to predict progression to DNC. Accurate early prediction of DNC may help avoid potentially inappropriate interventions, align treatment strategies with patient preferences and facilitate transparent family discussions. Moreover, in many countries, DNC is the legal basis for deceased organ donation, highlighting its ethical, organizational and societal relevance [4]. Despite the routine availability of clinical, neuroimaging and laboratory data in intensive care, the prognostic value of specific parameters for predicting DNC remains unclear.

Therefore, this systematic review and meta-analysis aimed to assess and synthesize the available evidence on clinical, neuroimaging, neurophysiological and laboratory predictors of progression to DNC in adults with non-traumatic acute brain injury, with the goal of informing early risk stratification and supporting decision-making in the intensive care setting. The review focused on non-traumatic causes of severe brain injury encountered in neurological and neurocritical care, including primary neurological and hypoxic-ischemic etiologies. Traumatic brain injury was considered outside the predefined scope because it is generally evaluated within a distinct trauma-specific clinical context.

## Methods

### Literature search and study selection

We performed a systematic literature search using MEDLINE and Embase (accessed via Ovid), Scopus, Web of Science Core Collection and Cochrane Central Register of Controlled Trials, spanning from database inception to August 31, 2026. Search strategies were constructed by combining controlled vocabulary and free-text terms related to acute brain injury, DNC and prognosis or prediction. These were structured using Boolean operators and tailored to each database’s syntax. In MEDLINE, text words “acute,” “severe,” or “critical” in combination with terms referring to brain, cerebral, intracranial or central nervous system injury or damage were used to identify studies addressing acute brain injury. These terms were combined with text words describing DNC or irreversible loss of brain function, as well as with prognostic concepts captured using the text words “predict*” and “prognostic*.” All text words were used in combination with their corresponding Medical Subject Headings (MeSH), including “brain Injuries” and “brain death”, to ensure coverage of relevant articles indexed in MEDLINE. The three concepts were combined using the Boolean operator “AND.” Scopus and Web of Science employed analogous terms in title, abstract, and keyword fields. The Cochrane Central Register used the same keyword structure. Database-specific search strings are detailed in the Supplementary Material (S1).

The reference lists of relevant reviews, meta-analyses and opinion articles were manually screened to identify additional eligible studies. All titles and abstracts retrieved through the database searches were screened for eligibility by two reviewers (DS and MN) independently. Full-text articles of potentially relevant studies were then assessed in detail to determine whether they met the predefined inclusion criteria. Disagreements between reviewers were resolved through discussion; if consensus could not be reached, a third reviewer (KB) was consulted. The review was conducted in accordance with the Preferred Reporting Items for Systematic Reviews and Meta-Analyses (PRISMA) guidelines [5]. This study was registered in PROSPERO (CRD42024537918) [6].

### Eligibility criteria

The study eligibility criteria were defined in terms of (1) Participants, which included adult patients with non-traumatic acute brain injury including hypoxic-ischemic encephalopathy, intracerebral hemorrhage, ischemic stroke and infectious or inflammatory diseases such as meningoencephalitis; (2) Index prognostic factors consisting of clinical findings (e.g., Glasgow Coma Scale scores, pupillary reflexes, and intracranial pressure), neuroimaging findings on CT and MRI, neurophysiological (e.g., somatosensory evoked potentials) and laboratory biomarkers (e.g., neuron-specific enolase), evaluated for their predictive value; (3) Comparators, comprising patients within the same study populations who did not progress to DNC; (4) Outcomes, defined as DNC reported as a clinical endpoint according to national or international standards; and (5) Study design including randomized controlled trials, prospective and retrospective cohort studies, case-control studies, cross-sectional studies, case series involving five or more patients and registry-based analyses. Studies limited to traumatic brain injury or pediatric populations, those assessing surrogate outcomes (e.g., Cerebral Performance Category scores or all-cause mortality without confirmed DNC), case reports, small case series and conference abstracts without full-text availability were excluded. Studies of mixed cohorts that also included patients with traumatic brain injury were eligible if non-traumatic etiologies were represented. No limitations were imposed on language or publication date.

### Data extraction

Data on the following items were extracted from the full-text articles of the included original studies and entered into a pre-specified Excel spreadsheet (Microsoft, Redmond, WA, USA): (1) study characteristics (first author, year of publication, study design, study population and study location); (2) prognostic parameters assessed, including clinical indicators (e.g., neurological examination findings), imaging features (e.g., CT or MRI findings) and laboratory biomarkers; (3) definition of DNC; (4) population characteristics, including the number of patients progressing to DNC relative to the total study population); and (5) adjustment for potential confounders.

### Risk of bias assessment

Risk of bias was assessed using the Quality in Prognosis Studies (QUIPS) tool [7]. The following domains were evaluated: study participation, study attrition, measurement of prognostic factors, measurement of outcomes, confounding and statistical analysis. Detailed criteria are provided in Supplementary Material 2. For each QUIPS domain, studies were classified as having a low, moderate, or high risk of bias. The overall risk-of-bias rating was subsequently derived from the domain-level assessments: studies were considered at low overall risk when all domains were rated as low risk, at high overall risk when at least one domain was rated as high risk, and at moderate overall risk for all remaining combinations of domain ratings.

### Data synthesis and statistical analysis

Statistical analyses were performed using STATA (version 16.1; StataCorp, College Station, TX, USA) and Python (version 3.13; Python Software Foundation). Where sufficient data were available, meta-analyses were conducted using random-effects models to account for anticipated clinical and methodological heterogeneity between studies. Between-study variance was estimated using the restricted maximum-likelihood (REML) method. For continuous outcomes, pooled estimates were calculated as mean differences (MDs) with corresponding 95% confidence intervals (95% CIs) using inverse-variance weighting. When continuous data were reported as median and interquartile range (IQR), the corresponding mean and standard deviation were estimated as previously reported [8]. For dichotomous outcomes, pooled odds ratios (OR) were calculated on the logarithmic scale and back-transformed for presentation. Statistical heterogeneity was assessed using Cochran’s Q test and quantified with the I² statistic. Studies were grouped according to the type of prognostic factor assessed (clinical and neurophysiological findings, neuroimaging markers, or laboratory biomarkers). Studies were included in the quantitative synthesis when comparable data for the same prognostic factor were available from at least two studies. Quantitative synthesis was performed only when studies were considered clinically and methodologically comparable with respect to the underlying patient population and the definition and assessment of the prognostic factor. Studies were required to report an equivalent outcome of DNC, although terminology and diagnostic criteria could vary between studies. Given the limited number of studies available for quantitative synthesis, meta-regression and formal subgroup analyses, and assessment of small-study effects or publication bias were not performed. All statistical tests were two-sided and a p value < 0.05 was considered statistically significant. The certainty of evidence for the prognostic factors included in the quantitative synthesis was assessed using the GRADE approach, adapted for prognostic-factor studies. The assessment considered five domains: risk of bias, inconsistency, indirectness, imprecision, and publication bias (Supplementary Material 3).

## Results

### Study selection and characteristics

**Fig. 1:**
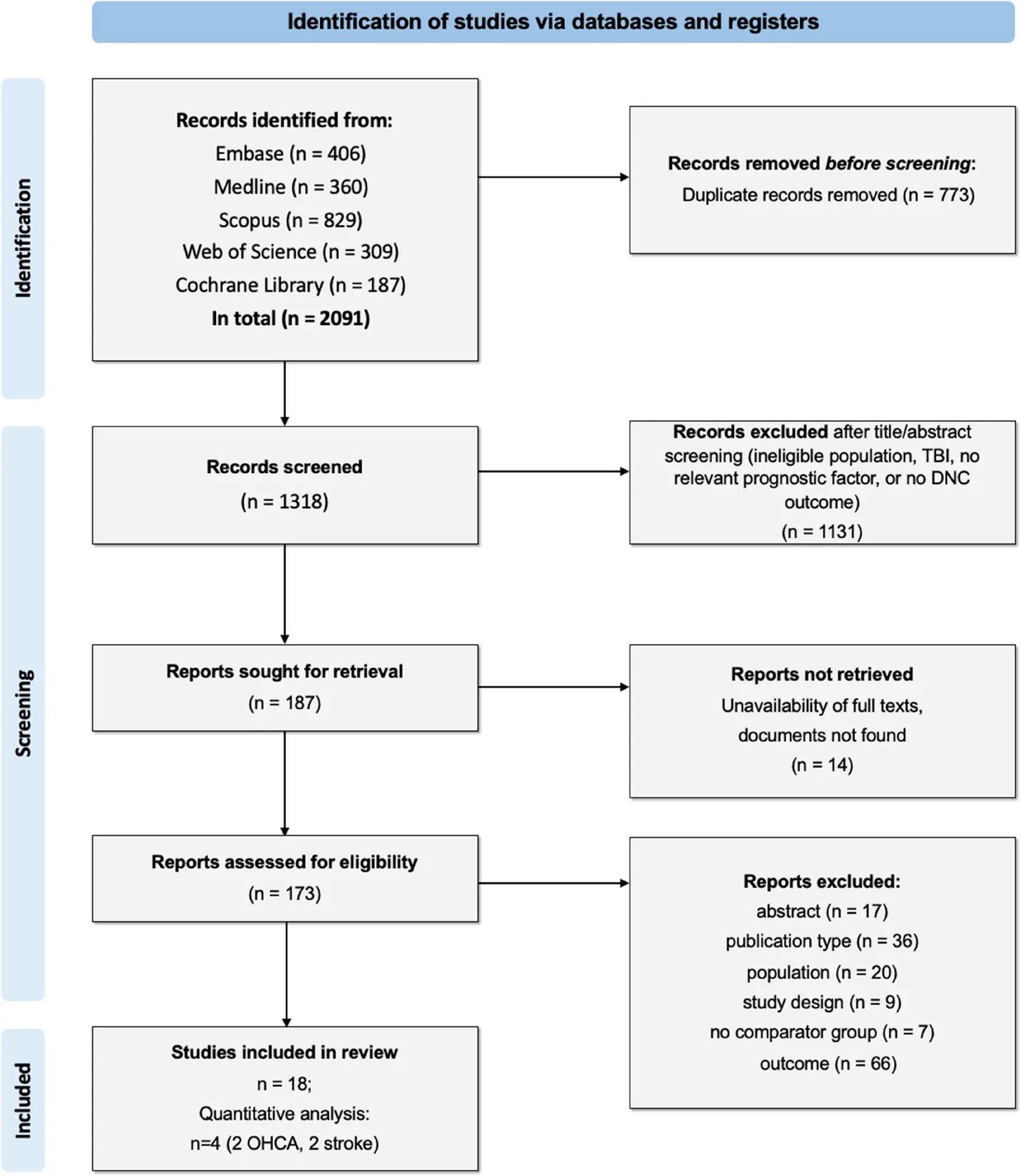
PRISMA flow-chart

As shown in the PRISMA flow diagram (Fig. 1), a total of 2,091 records were identified through electronic database searches and manual screening of reference lists. After removal of duplicates, 1,318 titles and abstracts were screened, of which 173 full-text articles were assessed for eligibility. Finally, 18 studies met the inclusion criteria, and four were retained for quantitative synthesis [9–26].

Across the included studies, the reported study cohorts comprised a total of 9,070 participants. Nine studies investigated patients following cardiac arrest, predominantly out-of-hospital cardiac arrest [9–16, 26], while two studies focused exclusively on patients with severe stroke [17, 18]. The remaining studies included heterogeneous populations with severe acute brain injury in neurocritical care settings [19–25]. The majority of studies were observational cohort studies, predominantly retrospective [9–18, 20, 21, 23], while a smaller number used prospective observational, diagnostic accuracy or prospective validation designs [19, 22, 24–26]. Most cohorts were single-center ICU-based, although some studies included external validation cohorts or multicenter populations [13, 17, 26].

Cohort sizes ranged from 18 patients [15] to 2,204 patients [11]. Reported rates of progression to DNC varied substantially according to study design and population. In neurocritical care screening cohorts, DNC occurred in 1.9% of patients [24], whereas incidences of approximately 9–29% were reported in post-cardiac arrest cohorts [9–14, 16, 26]. In cohorts with severe primary brain injury, including severe stroke, progression to DNC exceeded 20% in several studies [17, 22].

Definitions of DNC were generally consistent with accepted clinical and legal standards but varied in the requirement for ancillary testing. Several studies defined DNC according to national or professional guidelines or legislation [10–13, 16, 21, 23, 24, 26], while others did not further specify diagnostic criteria [15, 18, 20, 22, 25]. One study applied a study-specific definition [9], whereas another defined DNC within the context of organ procurement under French legislation [17].

### Reported predictors of progression to DNC

In post-cardiac arrest populations, computed tomography (CT)-based predictors were most frequently evaluated, primarily reflecting diffuse cerebral edema and hypoxic-ischemic injury [10–12]. Assessed imaging features included qualitative loss of gray-white matter differentiation as well as quantitative gray-white matter ratio (GWR) measurements [10–12]. Predefined GWR thresholds were applied in two studies, including a fixed cutoff of ≤ 1.23 [12] and time-dependent thresholds (< 1.10 and < 1.20) within the first 24 h after arrest [11]. Other studies evaluated qualitative CT signs of cerebral edema, such as sulcal effacement, basal cistern effacement or compression [12, 21, 22]. Additional CT predictors assessed in devastating brain injury cohorts included ventricular dilatation and radiological signs of herniation as well as midline shift [21, 22].

Two studies evaluated patients with severe stroke, one restricted to large ischemic infarction [17] and one comprising predominantly hemorrhagic stroke [16], and focused on CT predictors of progression to DNC. In these cohorts, imaging markers indicating severe intracranial mass effect - including herniation and effacement or absence of cerebrospinal fluid spaces - were identified as predictors of DNC [18]. One study additionally incorporated clinical and imaging parameters such as coma severity (GCS ≤ 6), radiological herniation, hydrocephalus, large stroke volume (> 65 mL), elevated admission systolic blood pressure (> 150 mmHg) and history of alcohol abuse into a multivariable prediction model [17].

Clinical and treatment-related predictors were examined primarily in post-cardiac arrest studies, often alongside imaging or neurophysiological variables [9, 14, 15, 26]. These included laboratory biomarkers (e.g., troponin, glucose, neuron-specific enolase), hemodynamic support requirements, arrest-related variables, temperature-management-related exposures and post-resuscitation hyperthermia (rectal temperature ≥ 38 °C) [9, 14, 15]. In one cohort, absence of pupillary light reflexes and elevated neuron-specific enolase within 6 h after return of spontaneous circulation were identified as independent predictors of DNC [16].

Neurophysiological predictors were evaluated in a limited number of studies and included EEG background suppression as part of a multimodal prediction model [12] and somatosensory evoked potential (SEP) patterns classified as absent, pathological or normal [23]. Additional physiological or surrogate predictors evaluated in single studies included optic nerve sheath diameter measured by transorbital ultrasound [19] and pituitary-axis hormone levels assessed at the time of DNC determination [20].

Three studies developed or evaluated dedicated screening or prediction tools for identification of patients at risk of DNC [13, 24, 26]. One study developed and validated the BDR score incorporating non-shockable rhythm, drug overdose etiology, CT signs of cerebral edema, FOUR score components and age < 45 years, using variables available within the first 24 h after cardiac arrest [13]. A second study developed and validated the BDCA score based on sex, initial rhythm, cardiac arrest etiology, serum sodium at 24 h and vasoactive drug use at admission and at 24 h [26]. Another study evaluated the clinical examination-based DETECT screening tool relying on coma and bilaterally absent pupillary light reflexes [24]. A detailed overview of reported predictors is provided in Table 1. We summarized the identified prognostic factors by category in Table 2, while dedicated screening tools and prediction scores are presented separately in Table 3.

**Table 1:** Details of included studies

Study (Author, Year)	Study design, population and location	DNC predictor(s) assessed	Definition of DNC	Factors associated with DNC	Factors not associated with DNC		Cases, *n*			Confounder adjustment	
**Bartos et al., 2018** [9]	Observational cohort study (retrospective); 12/2015-01/2018, 83 adults (77% men), mean age 56 years, refractory OHCA (VF/VT) treated with ECPR/ECMO; cardiac ICU (CICU); Minnesota, USA (single center)	Clinical/treatment variables (troponin level at 4 h post-admission, glucose level at 16 h post-admission; hemodynamic support / use of inotropes and pressors at 2–48 h); early neuro markers (CT evidence of anoxic injury/edema on admission, cerebral oximetry/NIRS within 24 h of admission, NSE on admission)	Absent brainstem reflexes and either herniation on head CT or nuclear brain perfusion scan demonstrating no cerebral flow	Elevated troponin level at 4 h post-admission; elevated glucose level at 16 h post-admission; greater hemodynamic support/use of inotropes and pressors at 24–48 h; declining cerebral oximetry/NIRS over the first 48 h; elevated NSE on admission; higher creatinine at 24 h. *Associated with poor neurological outcome*,* not DNC-specific*: anoxic injury/cerebral edema on CT	Absolute cerebral oximetry/NIRS values; S100B on admission		14/83 at CICU			Observational comparisons; no reported multivariable confounder	
**Case et al., 2024** [10]	Observational cohort study (retrospective); 01/2011-03/2020, 1,133 patients (60% men), mean age 58 ± 16 years; post-cardiac arrest cohort; Pittsburgh, USA (single center)	Early CT metrics (HU variance, mixture-model mean difference) from first CT (~ 4 h post-arrest) vs. GWR; edema (sulcal/basal cistern effacement on first CT)	Death by neurologic criteria (DNC) via American Academy of Neurology (AAN) guidelines	Lower GWR (AUC 0.82), lower HU variance (AUC 0.73), sulcal effacement (AUC 0.80), and basal cistern effacement (AUC 0.76) on first brain CT (median 4.2 h post-arrest)	Difference between mixture-model means on first brain CT (AUC 0.56; *p* = 0.38)		115/1,133			No (no multivariable adjustment); logistic regression included CT metrics only	
**Case et al., 2025** [11]	Observational cohort study (retrospective); 01/2010-12/2023, 2,204 patients (60% men), mean age 59 ± 16 years; comatose post-cardiac arrest cohort; Pittsburgh, USA (single center)	Time-dependent GWR thresholds on CT, stratified by hours from arrest (0–24 h)	Death by neurologic criteria (DNC) via American Academy of Neurology (AAN) guidelines	Reduced GWR on brain CT within 24 h post-arrest (GWR < 1.10 and < 1.20); strongest predictive sensitivity at 4–5 h post-arrest	None explicitly reported as not associated with DNC		248/2,204			No (no multivariable adjustment); time-stratified analyses only	
**Coppler et al., 2022** [12]	Observational cohort study (retrospective); 01/2010-02/2020, 1,569 patients (59% men), mean age 59 ± 16 years; comatose out-of-hospital cardiac arrest cohort; Pittsburgh, USA (single center)	Early brain CT 4.2 h post-arrest (sulcal effacement, low GWR ≤ 1.23) and early EEG background suppression ~ 9.5 h post-arrest	Death by neurologic criteria (DNC) via American Academy of Neurology (AAN) guidelines	Sulcal effacement on early CT; EEG background suppression combined with GWR ≤ 1.23	None explicitly reported as not associated with DNC			147/1,569		Yes (multivariable ridge regression including demographic, arrest, clinical, CT-GWR + sulcal/basal cistern effacement, and EEG parameters)	
**Dominguez-Roldan et al., 2004** [18]	Observational case–control study (retrospective CT review); 30 ICU patients with acute ischemic stroke (13 men [43%], 17 women; mean age 44.5 years), carotid or middle cerebral artery territory infarction, age 18–65 years, CT performed ≤ 24 h prior to ICU admission; Sevilla, Spain (single center neurological ICU)	CT signs on admission (insular ribbon sign, hyperdensities inside the big arteries of the base of the skull, hemispheric hypodensities, midline shift, and compression of the cerebrospinal fluid spaces)	Brain death (not further defined)	Midline shift; compression of the ambient cistern on admission CT	Insular ribbon sign; hyperdensity of the large basal cerebral arteries; hemispheric hypodensity on admission CT				15/30	No (univariate analyses only using chi-square, unpaired t-test, and Fisher exact test; no multivariable adjustment)	
**Humbertjean et al., 2016** [17]	Observational cohort study (retrospective); 01/2012-12/2013, 400 patients (47% men), mean age 68 ± 16 years; severe non-traumatic stroke (73% hemorrhagic) patients flagged via organ-procurement coordination; Lorraine region, France (five centres)	Initial admission variables (≤ 24 h post-stroke): GCS ≤ 6; herniation; hydrocephalus; initial systolic BP > 150 mmHg; history of alcohol abuse; stroke volume > 65 mL	Adjudicated brain death within the French organ procurement and transplant coordination system	Initial GCS ≤ 6; herniation; hydrocephalus; initial systolic BP > 150 mmHg; history of alcohol abuse; stroke volume > 65 mL	None explicitly reported as not associated with DNC				91/400	Yes (multivariable logistic regression including GCS, radiological herniation, hydrocephalus, stroke volume, systolic blood pressure, and alcohol abuse)	
**Karakitsos et al., 2006** [19]	Observational cohort study (prospective); 01/2003-03/2006, 54 patients (52% men), mean age 40 ± 19 years; severe brain injury cohort (GCS ≤ 8) with serial CT and transorbital ultrasound monitoring; Athens, Greece (single center)	Optic nerve sheath diameter (ONSD) measured by transorbital ultrasound at admission, ~ 12 days, and ~ 35 days (each with synchronous CT)	Clinical brain death (coma, absent brainstem reflexes, flat EEG, positive apnea test) with mandatory angiographic confirmation of absent cerebral blood flow (Hellenic state law)	ONSD > 5.9 mm and an increase ≥ 2.5 mm between serial measurements were associated with poor prognosis, but ONSD monitoring had low predictive value for brain death	No specific factor reported				22/54	No (unadjusted analyses; no multivariable adjustment reported)	
**Kitlen et al., 2023** [13]	Observational cohort study (retrospective); 2011–2021, development cohort: 362 patients (63% men), mean age 57 ± 16 years; validation cohort: 86 patients (71% men), mean age 52 ± 16 years; comatose out-of-hospital cardiac arrest cohort; United States (two academic centers)	BDR score: non-shockable rhythm (arrest), drug overdose etiology, early CT edema signs (≤ 24 h post-arrest), initial FOUR score (admission), age < 45 years.	Brain death via American Academy of Neurology (AAN) guidelines	Non-shockable rhythm; drug overdose etiology; loss of grey-white differentiation or sulcal effacement on CT within 24 h; FOUR score 0–5; age < 45 years. BDR score > 4: sensitivity 90%/88%, specificity 83%/65% in development/validation cohorts	No specific factor reported				58/362 (development cohort); 17/86 (validation cohort)		Yes (multivariable model; variables included in BDR score)
**Lee et al., 2022** [16]	Observational cohort study (retrospective analysis of prospectively collected data); 05/2018-02/2021, 108 adult patients (72.2% men), median age 55 years (IQR 41–69); comatose non-traumatic out-of-hospital cardiac arrest survivors treated with targeted temperature management (TTM); single academic center; Republic of Korea	Early absence of pupillary light reflex (4–6 h after ROSC) and initial serum neuron-specific enolase measured within 6 h after ROSC; and gray-to-white matter ratio (GWR) measured on brain CT within 6 h after ROSC	Brain death diagnosed according to Korean law using clinical examination, apnea testing, and EEG (± neuroimaging)	Initial serum NSE in the highest quartile and absent pupillary light reflex within 6 h after ROSC; combined AUC 0.96. GWR was assessed and was lower in patients progressing to BD (median 1.11 vs. 1.25; *p* = 0.006), but was not retained as an independent predictor.	No specific factor reported				31 / 108	Yes (multivariable logistic regression)	
**Madelaine et al., 2021** [25]	Development and validation study; development cohort: 569 patients alive 24 h after OHCA; validation cohort: 487 patients; multicenter cohorts, France.	Clinical and biological variables available within 24 h after OHCA; BDCA score components: sex, initial rhythm, cardiac or neurological cause of OHCA, serum sodium at 24 h, and vasoactive drug use at admission and at 24 h	Brain death, defined as irreversible loss of all brain function	BDCA Score: female sex; nonshockable rhythm; neurological cause of OHCA; higher serum sodium at 24 h; vasoactive drug use at admission and at 24 h, cardiac cause of OHCA (inverse)	None explicitly reported	84/569 (14.8%)				Multivariable prediction modeling using a binomial two-stage adaptive LASSO procedure; subsequent validation in an independent prospective multicenter cohort	
**Mazzeo et al., 2019** [20]	Post-hoc observational cohort study; patients recruited from previous prospective studies; 113 critically ill ICU patients; mixed critical illness cohort (ARDS *n* = 36, severe TBI *n* = 25, SAH *n* = 21, neurocritical patients at time of brain death *n* = 31); mean age 52.0 ± 16.6 years; 58.9% men; Italy (single university center)	Neuroendocrine (pituitary axis) hormone levels (fT3, fT4, cortisol, GH, prolactin, copeptin) measured on day 1, day 2–3, and day 4–5 after ICU admission	At the moment of Brain death (undefined)	Hormonal alterations assessed: fT3/fT4 (thyroid axis), cortisol (HPA axis), GH, prolactin and copeptin; thyroid-axis suppression and copeptin activation were the most frequent alterations overall	No specific factor reported				31/113	No (unadjusted, descriptive group comparisons)	
**Paul et al., 2025** [14]	Observational cohort study (retrospective); 01/2005-06/2021, 256 patients (65% men), median age 63 years (IQR 53–75); comatose post-cardiac arrest patients with severe anoxic brain injury who died from brain death or postanoxic encephalopathy; Versailles, France (single center ICU)	TTM (temperature-management) exposure (≥ 24 h), prolonged no-/low-flow > 30 min, neurological or hanging etiology, elevated PaCO₂ (days 1–2 post-admission)	Brain death via French legal criteria (clinical examination with coma, absent brainstem reflexes, positive apnea test, plus confirmatory CT angiography or two isoelectric EEGs)	No-flow + low-flow > 30 min; neurological cause of cardiac arrest or hanging; PaCO₂ >45 mmHg during days 1–2 after admission	TTM ≥ 24 h; shockable initial rhythm; maximal serum sodium > 143 mmol/L were not significantly associated in the multivariable analysis				56/256	Yes (multivariable regression adjusting for patient/arrest characteristics)	
**Ray et al., 2022** [21]	Observational cohort study (retrospective); 06/2014-09/2020, 205 patients; 164 patients confirmed dead using neurological criteria (DNC) and 41 patients with devastating brain injury who died following withdrawal of life-sustaining treatment; mean age 52 years (DNC) vs. 64 years (DBI); 50% men; devastating brain injury cohort; Bristol, United Kingdom (single center ICU)	CT signs on final pre-death brain CT (diffuse sulcal effacement, basal cistern effacement, loss of grey-white differentiation, contralateral ventricular dilatation)	Death by neurologic criteria (DNC) via UK Academy of Medical Royal Colleges’ Code of Practice (2008) with repeat clinical exams and apnoea testing by two clinicians	Loss of grey-white differentiation (46% DNC vs. 20% non-DNC); contralateral ventricular dilatation showed an inverse association (24% vs. 44%)	Brain herniation (79% vs. 76%); diffuse sulcal effacement; basal cistern effacement; midline shift > 5 mm				164/205	No (unadjusted comparisons only)	
**Revuelto-Rey et al., 2015** [22]	Prospective observational cohort study; 08/2011-05/2012, 166 patients (49.4% men), mean age 60.1 ± 21.8 years; acute severe brain injury patients undergoing head CT with midline shift > 5 mm and/or decreased or absent basal cisterns; Sevilla, Spain (single tertiary center)	CT on admission (emergency CT) as screening tool to identify potential BD donors (intracerebral haemorrhage, Glasgow Coma Scale < 8, and basal cistern alteration on head CT)	Brain death (not further defined)	Intracerebral hemorrhage, GCS < 8, and basal cistern alteration in univariate analysis; compression or absence of basal cisterns remained predictive in multivariable analysis	Midline shift > 5 mm was not identified as a significant predictor				34/166	Yes (multivariable analysis including basal cistern status, ICH, and GCS)	
**Scarpino et al., 2017** [23]	Observational cohort study (retrospective); 11/2014-10/2015, 203 patients initially identified, 194 analyzed; comatose patients with acquired brain injury of mixed aetiology (cardiac arrest, TBI, intracerebral haemorrhage, subarachnoid haemorrhage, brain infarction); age > 16 years; GCS ≤ 7 on admission; Florence, Italy (single tertiary center ICU)	SEP patterns (≤ 24 h post-ICU admission), classified as absent, pathological, normal	Brain death via Italian law (GCS = 3, absent brainstem reflexes, positive apnoea test, two isoelectric EEGs; ± TCD)	SEP grade 2 patterns (AP/AA) within 24 h of ICU admission; sensitivity 75.7%, specificity 76.6%	None explicitly reported				70/194	No (unadjusted analyses only)	
**Schoene et al., 2023** [24]	Prospective diagnostic accuracy study; 02/2020–01/2021, 414 patients (54.1% men), mean age 70.8 ± 14.8 years; consecutive adults admitted to neurocritical care with primary or secondary acute brain injury; Dresden, Germany (single tertiary neurocritical care center)	Automated digital screening tool (DETECT) based on coma and bilaterally absent pupillary light reflex, screened every 12 h during ICU stay	Brain death confirmed via German Medical Association guideline	Positive DETECT screening (coma + bilaterally absent pupillary light reflex), assessed every 12 h; sensitivity 100%, specificity 88.7%	Individual DETECT components were not separately evaluated as non-associated predictors				8/414	No (diagnostic accuracy study; unadjusted)	
**Takino & Okada, 1991** [15]	Observational cohort study (retrospective chart review); 01/1980-12/1990, 18 patients (44% men), median age 44 years (range 4–86); Post-cardiopulmonary resuscitation ICU cohort; Tokorozawa, Japan (single center)	Post-resuscitation hyperthermia (≥ 38 °C within 48 h post-ROSC; early onset ~ 6 h, peak > 39 °C at ~ 19.5 h)	Brain death (not further defined)	Hyperthermia > 39 °C within 48 h after resuscitation; patients progressing to BD also had earlier onset (median 6.2 h) and higher peak temperature (median 39.8 °C)	None explicitly reported				8/18	No (unadjusted analyses only)	
**Tejerina et al., 2023** [25]	Prospective observational cohort study; 11/2012-11/2020, 619 patients (62% men), mean age 62 ± 15.5 years; consecutive adult critically ill patients with structural brain injury (intracerebral hemorrhage, traumatic brain injury, subarachnoid hemorrhage, ischemic stroke) requiring ICU admission; Getafe, Spain (single tertiary ICU)	Age ≥ 60 years (baseline), low GCS ≤ 8 at ICU admission, intracerebral hemorrhage etiology at admission, intracranial hypertension during ICU stay	Brain death (not further defined)	GCS ≤ 8, intracranial hypertension, intracerebral hemorrhage, and age ≥ 60 years were predictors of unfavorable outcome (mRS 3–6)	Age ≥ 60 years was not significantly associated with brain death as cause of hospital mortality				68/619	Yes (multivariable adjustment for unfavorable outcome, not brain death)	

*Abbreviations*: AAN = American Academy of Neurology; BD = brain death; BDR = Brain Death Risk score; BP = blood pressure; CA = cardiac arrest; CICU = cardiac intensive care unit; CT = computed tomography; DBI = devastating brain injury; DNC = death by neurologic criteria; ECMO = extracorporeal membrane oxygenation; ECPR = extracorporeal cardiopulmonary resuscitation; EEG = electroencephalography; FOUR = Full Outline of UnResponsiveness score; fT3/fT4 = free triiodothyronine / free thyroxine; GCS = Glasgow Coma Scale; GH = growth hormone; GWR = grey-white ratio; HU = Hounsfield units; ICU = intensive care unit; ICH = intracerebral hemorrhage; IR = interquartile range; mRS = modified Rankin Scale; NIRS = near-infrared spectroscopy; NSE = neuron-specific enolase; ONSD = optic nerve sheath diameter; OHCA = out-of-hospital cardiac arrest; PaCO₂ = arterial partial pressure of carbon dioxide; PLR = pupillary light reflex; ROSC = return of spontaneous circulation; SAH = subarachnoid hemorrhage; SEP = somatosensory evoked potentials; TBI = traumatic brain injury; TCD = transcranial Doppler; TTM = targeted temperature management; VF/VT = ventricular fibrillation/ventricular tachycardia

**Table 2:** Summary of prognostic factors for DNC, grouped by category; specific patient populations are indicated in parentheses where applicable

Category	Associated with DNC	Not associated / inconsistent
**Imaging findings**	Reduced GWR *(OHCA)*; lower HU variance *(OHCA)*; sulcal effacement *(OHCA)*; basal cistern compression/effacement; loss of grey-white differentiation; herniation and hydrocephalus *(severe stroke)*; stroke volume > 65 mL *(severe stroke)*; contralateral ventricular dilatation *(inverse association)*	DWI *(OHCA)*; insular ribbon sign, basal artery hyperdensity and hemispheric hypodensity *(ischemic stroke)*; midline shift, herniation and sulcal/basal cistern changes showed inconsistent results across studies
**Clinical history / baseline / treatment**	Non-shockable rhythm *(OHCA)*; neurological cause/hanging *(post-cardiac arrest)*; drug-overdose etiology *(OHCA)*; prolonged no-/low-flow > 30 min *(post-cardiac arrest)*; female sex *(OHCA)*; cardiac cause *(inverse association; OHCA)*; age < 45 years *(OHCA*,* BDR-Score)*; SBP > 150 mmHg and alcohol abuse *(severe stroke)*; greater vasoactive/hemodynamic support *(OHCA)*	TTM ≥ 24 h *(post-cardiac arrest)*; shockable rhythm *(post-cardiac arrest)*; age ≥ 60 years
**Clinical examination**	Initial low GCS (≤ 6/≤8); low FOUR score *(OHCA)*; absent pupillary light reflex	Eye response and GCS motor score *(OHCA)*
**Biomarkers / laboratory**	Elevated NSE *(OHCA)*; S100B at 72 h *(OHCA)*; elevated troponin at 4 h and glucose at 16 h *(ECPR-OHCA)*; PaCO₂ >45 mmHg *(post-cardiac arrest)*; higher serum sodium at 24 h *(OHCA)*	S100B at admission/peak *(OHCA)*; NSE at 72 h *(OHCA)*; serum sodium finding**s** inconsistent across studies
**Other tests / monitoring**	Declining NIRS *(ECPR-OHCA)*; EEG background suppression + low GWR *(OHCA)*; SEP grade 2 patterns; hyperthermia > 39 °C *(post-resuscitation)*	Absolute NIRS *(ECPR-OHCA)*; SSEP N20 *(OHCA)*; SOFA-C *(OHCA)*; ONSD had limited DNC-specific predictive value

**Table 3:** Screening tools and prediction scores

Tool	Components / performance
BDR score	Rhythm, overdose etiology, CT edema, FOUR score, age; score > 4: sensitivity 88–90%, specificity 65–83%
**BDCA score**	Sex, rhythm, arrest etiology, serum sodium, vasoactive drug use; AUC 0.81–0.82
**DETECT**	Coma + bilaterally absent pupillary light reflex; sensitivity 100%, specificity 88.7%

### Quantitative data synthesis

Of the 18 included studies, four provided sufficiently comparable predictor definitions to allow for quantitative synthesis, comprising two severe stroke cohorts [17, 18] and two post-cardiac arrest cohorts [12, 16]. In the remaining studies, predictors were heterogeneous or assessed in isolation, precluding pooling across studies. Among the post-cardiac arrest studies, overlapping patient populations from the same institution were identified [10–12]; therefore, the most outcome-focused study was selected to avoid duplication of data [12].

In stroke populations, CT markers reflecting intracranial mass effect by means of unilateral absence of ambient cistern or herniation were associated with progression to DNC. Pooled analysis demonstrated a more than threefold increase in the odds of DNC associated with these imaging findings (pooled OR 3.14, 95%CI 1.98–4.96; *p* < 0.0001). No heterogeneity was observed between studies (I² = 0%, Q-test *p* = 0.95).

In post-cardiac arrest cohorts, patients who progressed to DNC had significantly lower GWR compared with those who did not. Random-effects analysis demonstrated a pooled mean difference of -0.15, 95%CI -0.22 to -0.07; *p* < 0.001, with substantial between-study heterogeneity (I² = 72%).

**Fig. 2:**
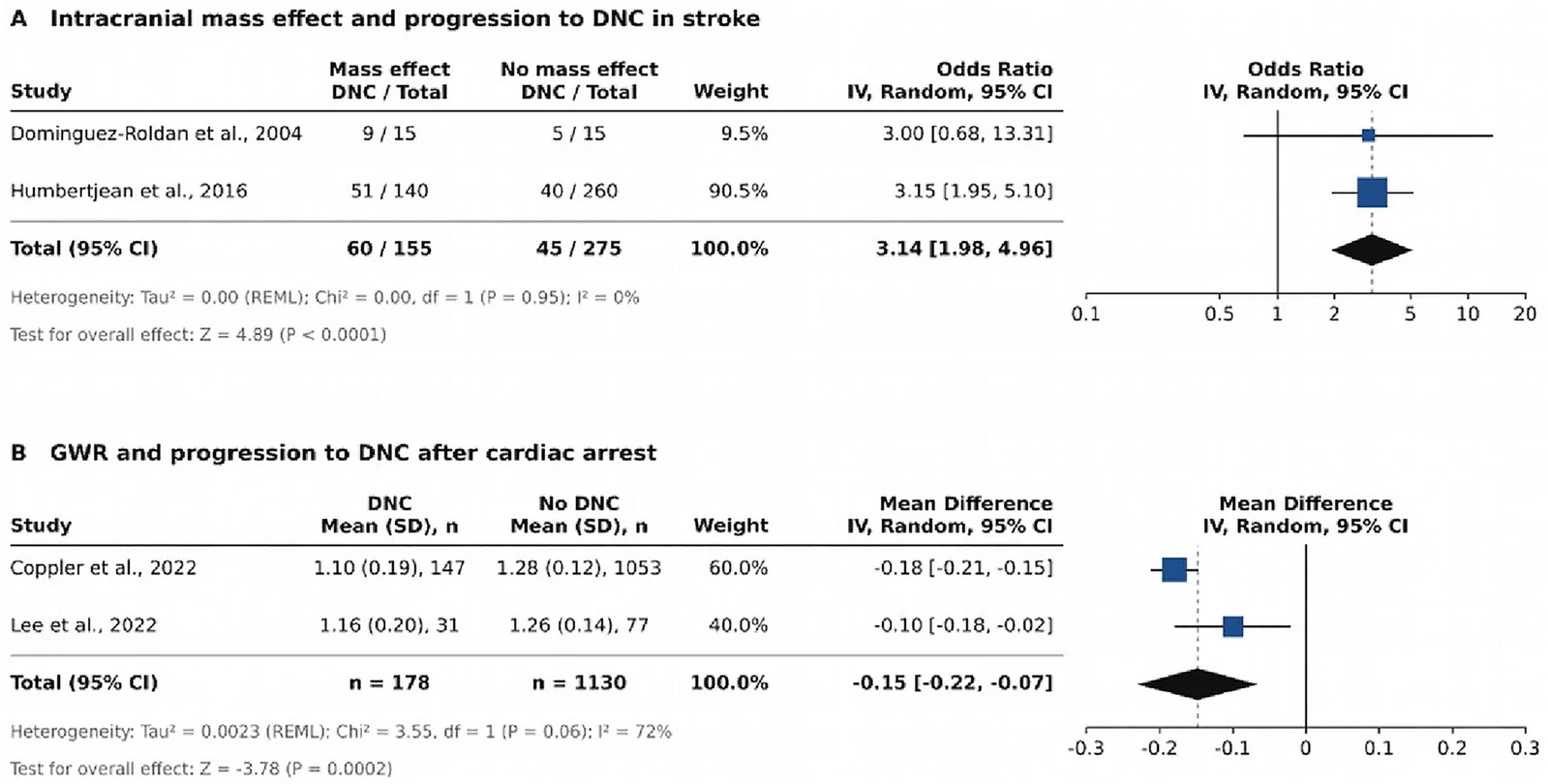
Forest plots showing pooled effect estimates for computed tomography-based predictors of progression to death by neurologic criteria. *Abbreviations:* CI, confidence interval; DNC, death by neurologic criteria; GWR, gray-white matter ratio; IV, inverse variance; MD, mean difference; REML, restricted maximum likelihood

Forest plots summarizing the meta-analysis are shown in Fig. 2. Given the small number of studies included in the meta-analysis, no subgroup or sensitivity analyses were performed. The certainty of evidence for these prognostic factors included in the quantitative synthesis is summarized in Table 4.

**Table 4:** GRADE assessment for prognostic factors associated with DNC; ¹Downgraded for risk of bias because both studies had concerns in several QUIPS domains, ²Downgraded one level for indirectness because the two studies evaluated different manifestations of intracranial mass effect in clinically distinct stroke populations ^3^Predominantly low risk of bias across QUIPS domains; Lee et al. had a concern in one domain, ^4^Downgraded for substantial statistical heterogeneity (I²=72%)

Prognostic factor	Studies	Pooled effect (95% CI)	Risk of bias	Inconsistency	Indirectness	Imprecision	Publication bias	Certainty
**CT markers for intracranial mass effect (severe stroke)**	Dominguez-Roldan et al.; Humbertjean et al.	OR 3.14, 95%CI 1.98–4.96; p<0.0001	Serious¹	Not serious (I²=0%)	Serious²	Not serious	Not assessed (2 studies)	Low ⨁⨁◯◯
**GWR (OHCA)**	Coppler et al.; Lee et al.	MD −0.15 (-0.22 to -0.07)	Not serious3	Serious4 (I²=72%)	Not serious	Not serious	Not assessed (2 studies)	Moderate ⨁⨁⨁◯

### Bias assessment

**Fig. 3:**
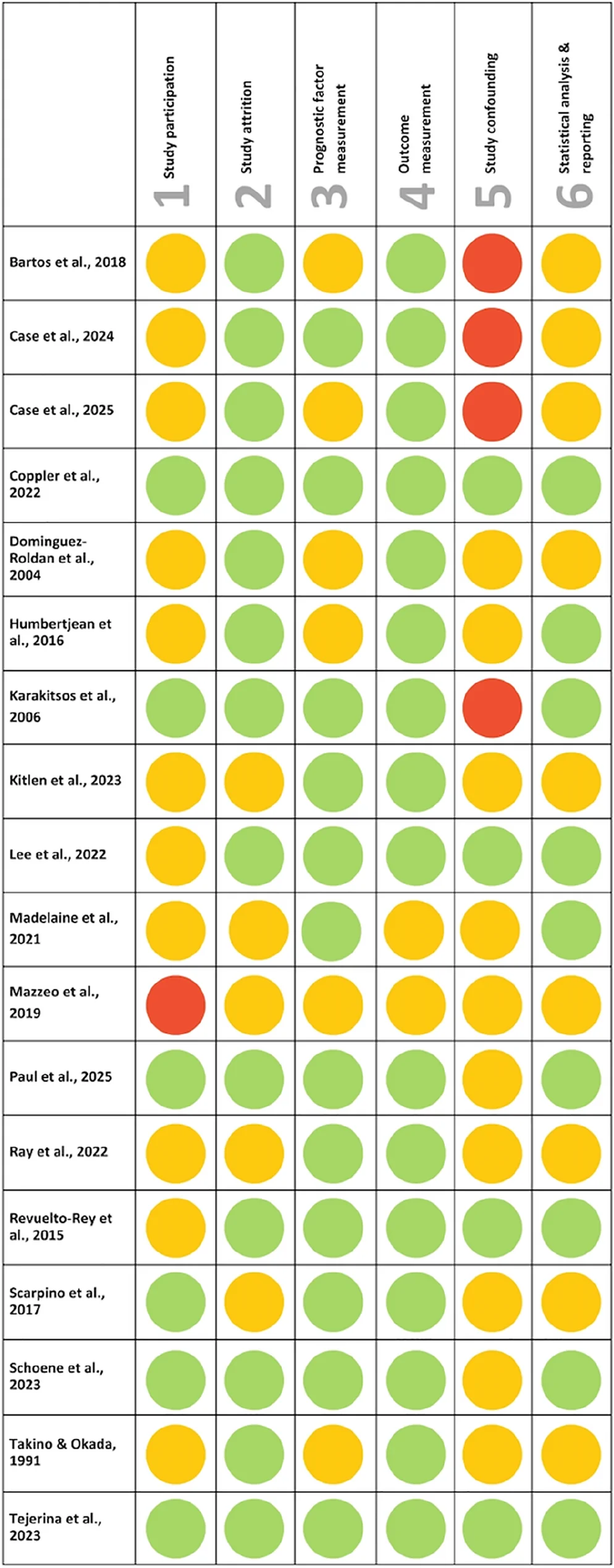
Risk of bias assessment. Green: low risk; yellow: moderate risk; red: high risk

Overall, five studies were rated as high risk of bias, eleven as moderate risk, and two as low risk of bias (Fig. 3). Assessment of small-study effects and publication bias was not performed, as fewer than ten studies were included in each meta-analysis, precluding meaningful interpretation of funnel plots.

## Discussion

In this systematic review and meta-analysis, we found that the existing evidence on predictors of DNC after non-traumatic acute brain injury is limited and methodologically heterogenous, with only a small number of studies assessing identical predictors in comparable patient populations, thereby allowing quantitative synthesis. Within these constraints, the most reproducible associations were observed for CT-based markers. In post-cardiac arrest populations, indicators of diffuse cerebral edema and loss of gray-white matter differentiation were associated with progression to DNC, whereas in stroke populations, early CT signs reflecting severe intracranial mass effect showed consistent associations with this outcome. These findings highlight the relevance of early structural brain injury in the pathophysiological trajectory toward irreversible loss of brain function.

In concordance with established neuroprognostication concepts, which emphasize global cerebral edema and impaired cerebral perfusion as key mechanisms in hypoxic-ischemic brain injury, patients exhibiting early radiological evidence of diffuse edema would be expected to be at particularly high risk of progressing to DNC. Both studies showed lower GWR values in patients progressing to DNC, although the magnitude of the association differed between studies [12, 16]. Consistent with our findings, a recent multicenter registry study identified a low GWR on early CT as an independent predictor of progression toward DNC after out-of-hospital cardiac arrest [27]; this study was not eligible for the present review because the outcome was operationally defined rather than based on formal determination of DNC. More broadly, early CT signs of global edema have been identified as strong predictors of poor neurological outcome in cardiac arrest survivors [12, 28].

However, the prognostic relevance of imaging findings was highly context-dependent and closely aligned with the underlying injury pattern and its temporal evolution. In post-cardiac arrest cohorts, prognostication was dominated by early global attenuation changes and CT signs of diffuse cerebral edema, reflecting widespread hypoxic-ischemic injury [10–12]. By contrast, in severe stroke populations, predictors of progression to DNC predominantly captured the development of life-threatening mass effects, with imaging markers such as brain herniation and obliteration of cerebrospinal fluid spaces showing the strongest associations [17, 18]. This pattern suggests that early CT markers do not serve as universal predictors but rather reflect distinct pathophysiological trajectories, with hypoxic-ischemic injury leading to rapidly progressive diffuse swelling, whereas severe stroke evolves from focal mass effect due to edema or hematoma toward secondary global compromise later in the disease course [17, 18].

Clinical and neurophysiological predictors provided additional but largely context-dependent prognostic information. In post-cardiac arrest cohorts, clinical findings, biochemical markers, and physiological parameters were associated with progression to DNC; however, their prognostic value varied substantially across studies and was highly susceptible to confounding by sedation, targeted temperature management, hemodynamic instability and ongoing life-sustaining therapies [9, 14, 16]. For example, absence of pupillary light reflexes and elevated neuron-specific enolase were independently associated with DNC in a single post-cardiac arrest cohort, underscoring both their potential relevance and the context-specific nature of these findings [16].

Neurophysiological measures, including EEG background suppression and somatosensory evoked potentials were associated with progression to DNC when profoundly abnormal. In a large post-cardiac arrest cohort, EEG background suppression combined with reduced GWR achieved a specificity of approximately 80% within a multimodal prediction model [12]. In patients with acquired brain injury of mixed etiology, bilaterally absent somatosensory evoked potentials were strongly associated with DNC, although sensitivity was limited and analyses were unadjusted [23]. Importantly, neurophysiological predictors were assessed in relatively few studies, often in specialized neurocritical care settings or at later stages of injury evolution, limiting applicability to early prognostication.

Despite these associations, none of the included studies identified a single predictor or combination of predictors that reliably and independently predicted progression to DNC. This finding aligns with established outcome-prediction frameworks in acute brain injury, which emphasize multimodal assessment and caution against early certainty when prognostic uncertainty persists [1, 2]. Accordingly, our results support current guideline recommendations advocating an integrated prognostic approach combining neuroimaging, clinical examination, neurophysiology and laboratory biomarkers, rather than reliance on any single parameter [28].

This study has several limitations. First, the available evidence was limited in quantity and methodologically heterogenous with substantial variability in patient populations, injury etiology, timing of predictor assessment and outcome definitions. Only a small number of studies provided data suitable for quantitative synthesis, restricting statistical power and precluding meaningful subgroup analysis or assessment of publication bias. Second, the majority of included studies were observational and retrospective, often single-center and frequently lacked adequate adjustment for confounders. In particular, clinical and physiological predictors were highly susceptible to confounding by sedation, targeted temperature management, hemodynamic instability and ongoing life-sustaining therapies, which may have influenced both predictor assessment and progression to DNC. Third, this review was deliberately restricted to non-traumatic acute brain injury. Traumatic brain injury was outside the predefined scope because it is generally evaluated within a distinct trauma-specific clinical context. Accordingly, our findings cannot be generalized to traumatic brain injury populations. Several mixed neurocritical care cohorts nevertheless included a subset of patients with traumatic brain injury, which were not analyzed separately. Fourth, the two stroke cohorts eligible for quantitative synthesis differed in stroke subtype: one included only large ischemic infarctions, whereas the other comprised predominantly hemorrhagic strokes. The pooled estimate for CT markers of mass effect therefore reflects severe stroke overall and subtype-specific analyses were not possible. Fifth, as in all prognostic studies in severe brain injury, the possibility of self-fulfilling prophecy and variability in end-of-life decision-making cannot be fully excluded.

## Conclusions

Evidence on prediction of progression to DNC after non-traumatic acute brain injury remains limited and methodologically heterogeneous. Within this available literature, CT-based markers appear to show the most reproducible associations with progression to DNC, reflecting diffuse cerebral edema and loss of gray-white matter differentiation after cardiac arrest and severe intracranial mass effect in stroke. Future research should focus on the prospective evaluation of standardized, multimodal prediction models to support early risk stratification for progression to DNC and informed clinical decision-making.

## Supplementary information

Below is the link to the electronic supplementary material.

Supplementary Material 1

## Data Availability

All analyzed data originate from published studies and are provided in the manuscript and supplementary material. Additional extraction tables and analysis scripts are available from the corresponding author upon reasonable request.

## Abbreviations

CI: Confidence intervals
CT: Computed tomography
DETECT: “screening for potential brain DEath in paTiEnts with severe brain damage and clinically asCerTained loss of cerebral functions”
DNC: Death by neurologic criteria
EEG: Electroencephalography
FOUR score: Full Outline of Unresponsiveness Score
GCS: Glasgow Coma Scale
GRADE: Grading of Recommendations Assessment, Development and Evaluation
GWR: Gray-white matter ratio
MD: Mean difference
MeSH: Medical Subject Headings
OR: Odds ratio
PRISMA: Preferred Reporting Items for Systematic reviews and Meta-Analyses
QUIPS: QUality In Prognosis Studies tool
REML: Restricted maximum-likelihood estimator
SEP: Somatosensory evoked potentials
