# Supplementary material for: Prediction of death by neurologic criteria in patients with non-traumatic acute brain injury: a systematic review and meta-analysis
Source: BMC Neurol. 2026 Oct 22;26:666. doi: 10.1186/s12883-026-05503-2 (PMC13692851; doi:10.1186/s12883-026-05503-2)
Supplement: Supplementary file 1 [file 12883_2026_5503_MOESM1_ESM.docx]

Early Prediction of Death by Neurologic Criteria in Patients with Non-traumatic Acute Brain Injury: A Systematic Review and Meta-analysis

Supplementary material

**Content**

Supplementary S1: Search strategy.....................................................................................................p.2

Supplementary S2: Risk of Bias Assessment......................................................................................p.5

Supplementary S3: Adapted GRADE framework for prognostic-factor evidence.............................p.7

**Supplementary S1**

**Search strategy for** **MEDLINE (Ovid platform)**

1. (severe or critical or acute) adj3 (brain or central nervous system or intracranial or cerebral or CNS) adj3 (damage or injury or lesion).mp.
2. exp brain Injuries/
3. Or/1-2
4. ((cerebral or brain* or neurologic* or encephalic) adj3 (death or dead or loss or cessation or arrest)).mp.
5. (irreversible adj3 (brain damage or brain injury or coma)).mp.
6. ((total or global or complete) adj3 (brain failure)).mp.
7. exp brain death/
8. or/4-7
9. (predict* or prognostic*).mp.
10. 3 and 8 and 9

**Search strategy for** **Embase (Ovid platform)**

1. (severe or critical or acute) adj3 (brain or central nervous system or intracranial or cerebral or CNS) adj3 (damage or injury).mp.
2. Exp brain injury/ or Exp brain damage/ or Exp nervous system injury/
3. or/1-2
4. ((cerebral or brain* or neurologic* or encephalic) adj3 (death or dead or loss or cessation or arrest)).mp.
5. (irreversible adj3 (brain damage or brain injury or coma)).mp.
6. ((total or global or complete) adj3 (brain failure)).mp.
7. Exp brain death/
8. or/4-7
9. (predict* or prognostic*).mp.
10. 3 and 8 and 9

**Search strategy for** **Scopus**

Search performed in Title, Abstract, and Keywords fields.

1. TITLE-ABS-KEY ((severe or critical or acute) W/3 (brain or “central nervous system” or intracranial or cerebral or CNS) W/3 (damage or injury))
2. TITLE-ABS-KEY((“brain injury”) or (“brain damage”) or (“severe brain injury”) or (“severe brain damage”))
3. 1 or 2
4. TITLE-ABS-KEY ((cerebral or brain* or neurologic* or encephalic) W/3 (death or dead or loss or cessation))
5. TITLE-ABS-KEY ((irreversible) W/3 (“brain damage” or “brain injury” or coma))
6. TITLE-ABS-KEY ((total or global or complete) W/3 (“brain failure”))
7. TITLE-ABS-KEY (“brain death”)
8. (4 OR 5 OR 6 OR 7)
9. TITLE-ABS-KEY (predict* or prognostic*)
10. (3) and (8) and (9)

**Search strategy for** **Cochrane Central Register of Controlled Trials (CENTRAL), via the Cochrane Library**

1. (severe or critical or acute) NEAR/3 (brain or central nervous system or intracranial or cerebral or CNS) NEAR/3 (damage or injury or lesion)
2. [mh brain diseases]
3. #1 or #2
4. (cerebral or brain* or neurologic* or encephalic) NEAR/3 (death or dead or loss or cessation or arrest)
5. irreversible NEAR/3 (brain damage or brain injury or coma)
6. (total or global or complete) NEAR/3 (brain failure)
7. [mh brain death]
8. #4 OR #5 OR #6 OR #7
9. predict* or prognostic*
10. #3 AND #8 AND #9

**Search strategy for Web of Science Core Collection**

1. TS=((severe or critical or acute) NEAR/3 (brain or “central nervous system” or intracranial or intracerebral or cerebral or CNS) NEAR/3 (damage or injury or lesion))
2. TS=((cerebral or brain* or neurologic* or encephalic) NEAR/3 (death or dead or loss or cessation or arrest))
3. TS=(irreversible NEAR/3 (“brain damage” or “brain injury” or coma))
4. TS=((total or global or complete) NEAR/3 (“brain failure”))
5. #1 OR #2 OR #3 OR #4
6. TS=(predict* or prognostic*)
7. #5 AND #6

**Supplementary S2**

**Risk of bias assessment using Quality in Prognosis Studies (QUIPS) tool**

Risk of bias was assessed using the QUIPS tool, adapted to studies evaluating early predictors of death by neurologic criteria (DNC) in adults with severe non-traumatic acute brain injury. Each domain was rated as follows:

**1. Study Participation**

*Low risk:* Study population representative of patients with severe brain injury at risk of DNC; clearly defined eligibility criteria; consecutive or complete inclusion.

*Moderate risk:* Minor concerns regarding representativeness or selection criteria.

*High risk:* Non-representative sample or selection bias likely to influence the relationship between predictors and DNC.

**2. Study Attrition**

*Low risk:* Minimal or non-differential loss to follow-up; missing data unlikely to affect the relationship between predictors and DNC.

*Moderate risk:* Some attrition with limited reporting or potential influence on results.

*High risk:* Substantial or unequal loss to follow-up likely related to predictors or DNC and not appropriately addressed.

**3. Prognostic Factor Measurement**

*Low risk:* Predictors clearly defined and measured using standardized methods within an early time window.

*Moderate risk:* Minor inconsistencies in measurement methods or timing.

*High risk:* Poorly defined or non-standardized predictor assessment likely to differ depending on outcome.

**4. Outcome Measurement (DNC)**

*Low risk:* DNC clearly defined and determined using standardized procedures.

*Moderate risk:* Outcome definition adequate but procedures insufficiently described or potentially variable.

*High risk:* DNC determination insufficiently defined

**5. Study Confounding**

*Low risk:* Important clinical confounders measured and appropriately adjusted for in the analysis.

*Moderate risk:* Partial consideration or incomplete adjustment for relevant confounders.

*High risk:* Key confounders not addressed.

**6. Statistical Analysis and Reporting**

*Low risk:* Appropriate statistical models and reporting of all results.

*Moderate risk:* Some methodological limitations or incomplete reporting.

*High risk:* Inadequate or inappropriate analyses likely to bias effect estimates.

**Supplementary S3**

**Adapted GRADE framework for prognostic-factor evidence**

**Based on the adapted GRADE framework proposed by Huguet et al. for prognostic-factor research.**

*Reference:* Huguet A, Hayden JA, Stinson J, McGrath PJ, Chambers CT, Tougas ME, Wozney L. Judging the quality of evidence in reviews of prognostic factor research: adapting the GRADE framework. Syst Rev. 2013 Sep 5;2:71. doi: 10.1186/2046-4053-2-71. PMID: 24007720; PMCID: PMC3930077.

| Domain | Criteria for downgrading (adapted from Huguet et al.) |
| --- | --- |
| Study limitations / Risk of bias | Downgraded when methodological limitations of prognostic-factor studies may have influenced effect estimates. |
| Inconsistency | Downgraded when prognostic-factor effects were inconsistent across studies and heterogeneity could not be explained. |
| Indirectness | Downgraded when populations, prognostic factors, outcomes, or settings differed from the review question. |
| Imprecision | Downgraded when estimates were based on few studies, small sample sizes, or wide confidence intervals. |
| Publication bias | Downgraded when selective publication was suspected. |

**Adaptations applied in the present review:**
Risk of bias was assessed using the QUIPS tool. No upgrading was performed based solely on effect magnitude because included evidence consisted of observational prognostic-factor studies. The phase-of-investigation domain proposed by Huguet et al. was not used for downgrading because included studies evaluated established prognostic factors in clinical cohorts rather than exploratory biomarker studies. Certainty ratings were categorized as high (⨁⨁⨁⨁), moderate (⨁⨁⨁◯), low (⨁⨁◯◯), or very low (⨁◯◯◯).
